# Application of bifidobacterium in tumor therapy

**DOI:** 10.3389/fonc.2025.1551924

**Published:** 2025-05-15

**Authors:** Yinwu Kong, Han Bai, Feifei Deng, Yaomin Zhao, Qianyan Li, Li Chang

**Affiliations:** ^1^ Yunnan Cancer Hospital, The Third Affiliated Hospital of Kunming Medical University, Peking University Cancer Hospital Yunnan, Kunming, China; ^2^ The 920th Hospital of Joint Logistics Support Force, Kunming, China

**Keywords:** bifidobacterium, malignant tumors, nanomaterials, tumor hypoxic microenvironment, tumor targeting, immune activation

## Abstract

Current clinical cancer treatments primarily rely on surgery, chemotherapy, radiotherapy, and immunotherapy; however, each approach has inherent limitations. In recent years, nanomaterials have gained significant attention in oncology due to their advantages in precise drug delivery, enhanced targeting, and improved therapeutic efficacy. Nevertheless, their clinical application remains limited by challenges such as complex synthesis, high costs, low delivery efficiency, and poor biodegradability. Bifidobacterium (BBM), a clinically used probiotic, has demonstrated unique tumor-targeting potential due to its obligate anaerobic nature, allowing it to selectively colonize, proliferate, and expand within the hypoxic tumor microenvironment. Recent advancements in synthetic biology and bacterial engineering have enabled the modification of Bifidobacterium as a microrobot for molecular imaging, drug or gene delivery, and other therapeutic functions. Compared to nanomaterials, Bifidobacterium-based bacterial therapy holds promise in overcoming certain limitations while potentially enhancing comprehensive cancer treatment by modulating the tumor microenvironment and boosting host immune responses. This review summarizes the latest progress in Bifidobacterium-mediated tumor imaging and therapy, explores its mechanisms of action, engineering strategies, and clinical applications, and discusses future directions for optimizing its functional design to improve therapeutic efficacy and safety.

## 1 Background

Cancer remains one of the most significant global health challenges. Currently, standard clinical treatments primarily include surgery, chemotherapy, radiotherapy, and immunotherapy (1–4). Despite continuous advancements in these conventional approaches, they still have inherent limitations. Surgical treatment often struggles to achieve complete cancer cell eradication, leading to a high risk of recurrence and metastasis, and its efficacy against metastatic tumors remains limited. While chemotherapy and radiotherapy are effective in eliminating cancer cells, they also damage healthy tissues, resulting in severe side effects. Additionally, the complexity of the tumor microenvironment (TME) can contribute to the development of resistance during prolonged chemotherapy and radiotherapy, such as chemoresistance and radioresistance, ultimately diminishing therapeutic efficacy (5–8). Although immunotherapy has introduced new possibilities for cancer treatment, it may still cause immune-related adverse effects, such as cytokine storms.

With the rapid advancement of nanotechnology, various nanoparticle-based strategies have been developed to enhance the efficacy of comprehensive cancer therapy.These strategies include polymeric nanoparticles (9–11), metallic or magnetic nanoparticles (12, 13), carrier-free nanoparticles, and size-tunable nanoparticles. Typically, these nanoparticles undergo complex synthesis and construction processes to achieve multifunctionality, enabling them to improve cancer treatment outcomes through precise drug delivery (14), photothermal and photodynamic therapy (15), radiosensitization (16), immune modulation (17), induction of tumor cell apoptosis or pyroptosis (18, 19), and regulation of the tumor immune microenvironment to overcome drug resistance (20) (21). Despite significant progress in enhancing targeted drug delivery to tumor sites, nanoparticle-based strategies still face several critical challenges, including complex synthesis processes, high production costs, and low efficiency (22). Moreover, synthetic nanoparticles often exhibit low biodegradability, poor stability, and potential toxicity, further limiting their clinical applications (23).

Bifidobacterium is an important symbiotic probiotic in the human gut, classified as a Gram-positive, obligate anaerobe. It is naturally non-pathogenic and non-toxic, and studies have demonstrated its potential antitumor effects and immunoregulatory functions (24). In recent years, Bifidobacterium has attracted significant attention in the field of cancer therapy due to its unique biological properties. The growth of malignant tumors is often accompanied by abnormal angiogenesis, where the resulting pathological vascular structures are highly disorganized and functionally defective. This leads to insufficient blood perfusion within the tumor, causing an inadequate supply of oxygen and nutrients to rapidly proliferating tumor cells. Such an imbalance in oxygen supply ultimately results in localized tumor hypoxia, forming the tumor hypoxic microenvironment (THME) (25). The THME not only influences tumor cell metabolism and invasiveness but also reduces the efficacy of conventional radiotherapy, chemotherapy, and immunotherapy. Consequently, targeting the THME has become a major focus in recent cancer research. Due to its obligate anaerobic nature, Bifidobacterium preferentially colonizes and proliferates in hypoxic regions of the body, making it an ideal candidate for targeting solid tumors (26, 27). This characteristic not only enables Bifidobacterium to exhibit high selectivity within the tumor microenvironment but also provides an innovative strategy for bacteria-based cancer therapy.

**Figure 1:**
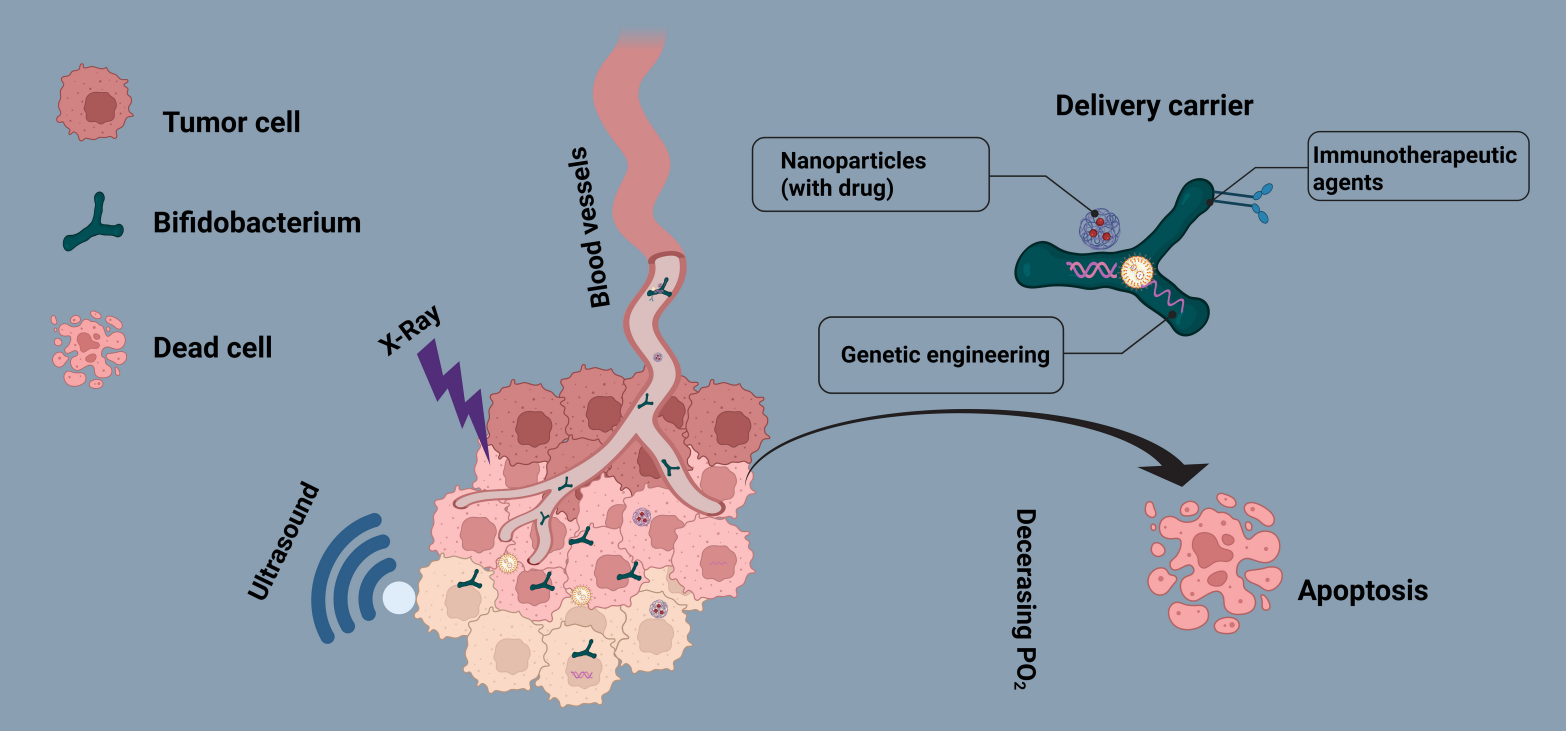
Mechanism of Bifidobacterium (BBM)-Mediated Comprehensive Cancer Therapy. The schematic illustrates the preferential colonization of Bifidobacterium within the tumor hypoxic microenvironment and its role as a carrier for delivering anticancer drugs or integrating with nanoparticles. Additionally, genetically engineered BBM can express exogenous genes to enhance tumor-targeted therapy, enabling tumor imaging, immune modulation, and multimodal synergistic treatment.

In this review, we systematically explore the application of Bifidobacterium in various anticancer therapies, including chemotherapy, targeted therapy, focused ultrasound ablation (FUA), radiotherapy (RT), and immunotherapy (**Figure 1**). Bifidobacterium not only serves as a natural delivery system for directly transporting chemotherapeutic agents, targeted drugs, or immunotherapeutic agents (such as PD-L1 inhibitors) but can also be combined with nanoparticle-based drug carriers to enhance drug accumulation at tumor sites, improve antitumor efficacy, and enable *in vivo* imaging and real-time monitoring. Furthermore, with advancements in synthetic biology and genetic engineering, Bifidobacterium can be engineered to express therapeutic proteins or reporter genes, allowing for more precise therapeutic and diagnostic functions within the tumor microenvironment. For instance, engineered Bifidobacterium can be designed to secrete antitumor cytokines, enhance host immune responses, or function as biosensors for dynamic tumor monitoring.In recent years, Bifidobacterium-based tumor therapy has demonstrated great potential in the field of personalized medicine. Through targeted modifications and optimizations, future developments may lead to safer, more efficient, and highly precise bacterial therapy strategies, offering innovative approaches for comprehensive cancer treatment.

## 2 Characteristics of bifidobacterium and the tumor microenvironment

Bifidobacterium is an essential component of the human gut microbiota, including species such as B. infantis, B. longum, and B. breve (28, 29). As a critical physiological bacterium in the human gut, Bifidobacterium is a Gram-positive, obligate anaerobe with no pathogenicity or toxicity toward the host. It also exhibits antitumor and immune-enhancing properties (30). Hypoxic regions are characteristic features of rodent models and various human solid tumors (31). Oxygen levels within most solid tumors vary, ranging from near anoxic (0% oxygen) to hypoxic (1% or 7.5 mmHg oxygen) and normoxic (8% or 60 mmHg oxygen), whereas normal tissues typically have oxygen levels between 24 and 66 mmHg (32). These hypoxic regions within solid tumors provide a favorable environment for the growth and proliferation of certain bacteria (33). Additionally, the ischemia and tissue hypoxia within tumors form an immunosuppressive microenvironment that further protects bacterial survival (34, 35). Consequently, anaerobic bacteria like Bifidobacterium and Lactobacillus, due to their ability to selectively colonize tumor tissues within the hypoxic and immunosuppressive tumor microenvironment, have been widely used as tumor-specific drug delivery carriers (36).

**Figure 2:**
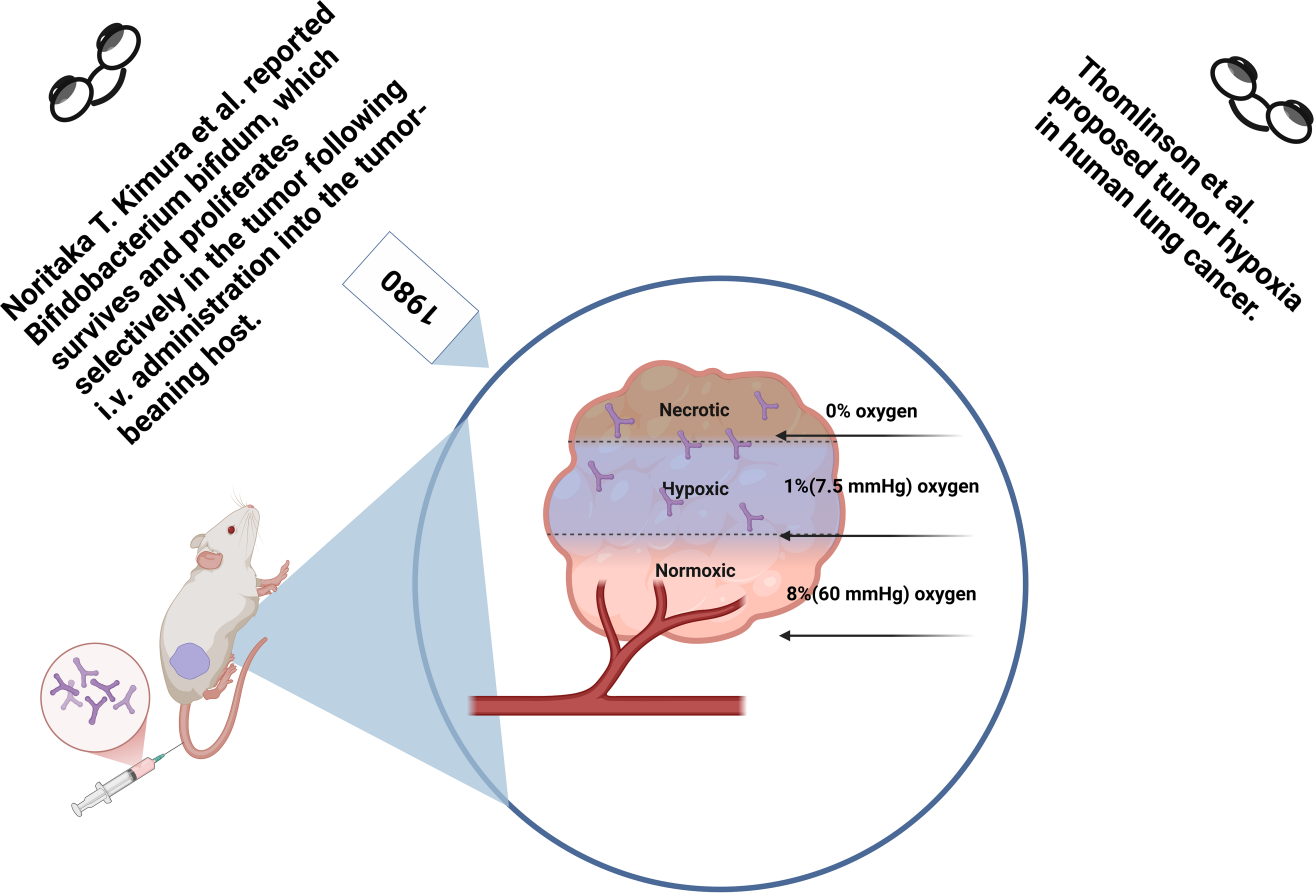
Safe colonization of bifidobacterium in tumor hypoxic regions after intravenous injection in mice. Bifidobacterium injected via the tail vein selectively accumulates in the tumor hypoxic microenvironment, rather than in normal tissues, demonstrating its excellent biosafety and tumor-targeting ability. This provides strong support for Bifidobacterium-based tumor therapies.

Tumor lesions are often accompanied by necrotic foci and hypoxic regions. When anaerobic bacteria are intravenously injected into tumor-bearing mice, they can freely proliferate in these hypoxic areas (37, 38), while avoiding healthy tissues with adequate blood flow and oxygen supply. The selective colonization of these bacteria within tumor tissues serves as a marker to distinguish tumors from normal tissues, potentially aiding in tumor diagnosis and treatment. As early as 2000, K. Yazawa and colleagues injected two strains of Bifidobacterium (105-A and 108-A) intravenously into B16-F10 melanoma and Lewis lung cancer mouse models (26). The results of quantitative culture experiments at 1, 24, 48, 72, 96, and 168 hours post-injection showed a significant increase in the number of B. longum within the tumor tissues, while the bacterial count in normal tissues (such as the lung, liver, spleen, kidney, and heart) gradually decreased over time and was nearly undetectable at 168 hours. Histological examination using Gram staining further confirmed these findings. In a similar study conducted 21 years later, researchers also observed that Bifidobacterium selectively proliferated in tumor tissues after intravenous injection, while its quantity in other organs rapidly declined, a result also confirmed by Gram staining (27, 39). Overall, researchers concluded that after intravenous injection into tumor-bearing hosts, Bifidobacterium could selectively survive and proliferate within tumor tissues, demonstrating excellent biosafety and targeting characteristics, making it a promising tool for tumor diagnosis and selective treatment (**Figure 2**). Consequently, Bifidobacterium has been utilized for gene, enzyme, drug, or nanoparticle delivery, in combination with focused ultrasound (FUA/HIFU), chemotherapy, radiotherapy, or immunotherapy, to enhance the diagnosis and treatment of solid tumors (23, 40).

## 3 Application of bifidobacterium combined with focused ultrasound in tumor imaging and treatment

7High-intensity focused ultrasound (HIFU) tumor ablation relies on the use of synergistic agents and precise imaging guidance. However, current synergistic agents often suffer from poor targeting ability and limited imaging capability, which restricts the therapeutic potential of HIFU (41–44). To enhance multimodal imaging and therapeutic efficacy, Wang et al. selected Bifidobacterium longum (B. longum) as a carrier to deliver the synergistic agent (CL-ICG-PFH-NPs) into solid tumors (39). Initially, B. longum was injected intravenously, taking advantage of its specific proliferative ability within tumor tissues to serve as a target for nanoparticles. After 7 days, B. longum had proliferated sufficiently within the tumor tissue. Then, CL-ICG-PFH-NPs were injected, where the electrostatic attraction between the cationic lipid nanoparticles and the negatively charged surface of B. longum facilitated the accumulation of nanoparticles in the tumor area. This approach enabled targeted multimodal imaging, HIFU enhancement, and real-time monitoring of the HIFU treatment process. Additionally, Tang et al. utilized the tumor-targeting ability of B. longum to deliver AP-PFH/PLGA NPs to the tumor area, promoting efficient HIFU cancer treatment.

In addition, due to the negatively charged surface of Bifidobacterium, it is expected to guide the accumulation of cationic nanoparticles (NPs) in tumor-targeted regions via electrostatic adsorption, without altering its physiological characteristics, thereby enabling biological targeting. Consequently, Wang et al. developed a biologically targeted oxygen-generating probe composed of Bifidobacterium—which naturally targets hypoxic tumor regions—and multifunctional oxygen-generating nanoparticles loaded with IR780, perfluorohexane (PFH), carboplatin (CBP), and oxygen. These probes are expected to achieve targeted and synergistic focused ultrasound ablation surgery (FUAS) therapy and dual-modal imaging, facilitating tumor diagnosis and treatment. IR780 is a lipophilic cationic compound with excellent fluorescence (FL) and photoacoustic (PA) imaging capabilities. As it does not require ligand modification, IR780 inherently possesses tumor-targeting properties, making it an ideal imaging dye. Compared to ultrasound imaging, which is commonly used in FUAS image monitoring, FL and PA imaging offer higher sensitivity and resolution, enabling visualization of tumor-targeted regions and assessment of the *in vivo* distribution dynamics of NPs—effectively addressing the limitations of current imaging techniques (45).

## 4 Bifidobacterium combined with chemotherapy and gene therapy

Since the late 1940s, various chemotherapy drugs have been used alone, in combination, or in conjunction with traditional surgery and radiotherapy (46). Despite advancements in newer treatment strategies such as targeted therapy and immunotherapy in recent years, chemotherapy remains a crucial approach in cancer treatment, both as a standalone treatment and when combined with other therapies (47). However, most chemotherapy drugs lack targeted delivery capabilities and may cause toxic effects on other organs, such as inducing intestinal mucositis, ultimately affecting the efficacy of chemotherapy (48). To address chemotherapy-induced intestinal mucositis and the resulting gut microbiome imbalance, Xu et al. conducted a quantitative analysis of the gut microbiome in gastric cancer patients before and after chemotherapy to assess the impact of Bifidobacterium intervention on microbiome balance (49). The results indicated that Bifidobacterium promoted the growth of beneficial bacteria, regulated microbiome imbalance, and prevented chemotherapy-induced dysbiosis, significantly reducing adverse drug reactions and improving patient tolerance to chemotherapy.

Additionally, Bifidobacterium has been used as a delivery system for tumor necrosis factor-related apoptosis-inducing ligand (TRAIL) and endostatin, which synergize with chemotherapy drugs to inhibit hypoxic tumors (50). In this application, Bifidobacterium, serving as a carrier, utilizes its unique biological targeting ability to colonize the S180 sarcoma. When combined with low-dose doxorubicin, Bifidobacterium inhibits the growth of S180 sarcoma, suggesting that delivering anticancer genes alongside low-dose chemotherapy drugs or other targeted genes using Bifidobacterium represents a promising strategy for tumor gene therapy.

The combination of Bifidobacterium with chemotherapy and gene therapy holds the potential for synergistic effects. Bifidobacterium can serve as a carrier for both chemotherapy drugs and gene therapy agents, facilitating their targeted delivery to tumor sites. This approach could not only enhance the cytotoxic effects of chemotherapy but also promote the targeted delivery of therapeutic genes to cancer cells. Despite the many potential advantages of this strategy, challenges remain, such as ensuring the safety of using live bacteria alongside chemotherapy and gene therapy. Furthermore, to translate this combination therapy into clinical practice, extensive preclinical and clinical studies are required to confirm its efficacy and safety. Regulatory approval and the development of personalized treatment strategies will be key factors for its successful clinical application.

## 5 Bifidobacterium in radiotherapy

Radiotherapy (RT) is one of the most widely used cancer treatment methods and is effective against various types of tumors (51). However, due to the rapid growth of solid tumors, oxygen supply is often insufficient, leading to the formation of a hypoxic microenvironment within tumors (52). This further reduces the sensitivity of cancer cells to radiotherapy (53–55). To combat radiotherapy resistance, various radiotherapy sensitizers have been developed (56–58); however, these sensitizers often face limitations, such as low targeting specificity, limited biological safety, high cost, and difficulty in procurement (59). Certain anaerobic bacteria, including Clostridium and Bifidobacterium, are known to selectively germinate and proliferate in the hypoxic regions of solid tumors after intravenous injection (37, 60). To use bacteria as carriers for radiotherapy sensitizers, a previous study utilized a Lewis mouse lung cancer model and combined Bifidobacterium and its specific monoclonal antibody (mAb) with radiotherapy (RT) for tumor treatment. By setting up different experimental groups, the researchers investigated the role of Bifidobacterium in tumor sensitization. They found that Bifidobacterium effectively colonized and grew within the tumor. When combined with radiotherapy and its specific monoclonal antibody, it showed significant therapeutic efficacy. This combination therapy not only delayed tumor growth to the greatest extent but also extended the overall survival of the treated mice (61). Moreover, on day 12 after treatment, three mice from each group were randomly selected, and their heart, liver, spleen, lung, and kidney tissues were subjected to hematoxylin and eosin (H&E) staining to evaluate potential side effects on surrounding tissues. The results showed that the Bifidobacterium injected via tail vein did not cause damage to vital organs, highlighting its high safety.

The combination of Bifidobacterium and radiotherapy as a potential strategy to enhance cancer treatment shows promising prospects. However, further research and development are needed to comprehensively explore and validate the synergistic effects of this combination therapy, with the ultimate goal of improving treatment outcomes and advancing personalized and targeted cancer therapies.

## 6 Bifidobacterium in immunotherapy

Cancer immunotherapy aims to destroy tumors by activating the immune system. It has become an effective treatment method alongside surgery, radiotherapy, chemotherapy, and targeted therapy. In a 2018 study, researchers analyzed the gut microbiomes of 42 melanoma patients and compared those who had a good response to immune checkpoint inhibitors (ICIs) with those who did not respond. 16S sequencing results showed that in the responders group, the abundance of Bifidobacterium was higher, suggesting that Bifidobacterium could regulate anti-tumor immunity and influence the efficacy of ICIs (62). Interestingly, 15 years ago, the team led by Thomas Gajewski observed that after subcutaneously transplanting B16.SIY melanoma cells into two different C57 mouse strains (Tac: C57BL/6NTac from Taconic Farms; Jax: C57BL/6NTac from Jackson Laboratory), the two strains exhibited significantly different immune responses, indicating that there were spontaneous anti-tumor immune differences between these strains (63). After tumor transplantation, the levels of IFN-γ in the spleen cells of the Jax mice were significantly higher than those in Tac mice, and there were more tumor-specific CD8+ T cells in the tumor, indicating that the differences in melanoma growth between the two mouse strains were immune-mediated. Interestingly, when the two strains were co-housed for three weeks prior to tumor transplantation, the anti-tumor differences disappeared, suggesting that the differences in gut microbiome composition were the primary cause of the immune differences. Further bacterial species phylogenetic analysis revealed significant differences in Bifidobacterium abundance.

In addition, Se-Hoon Lee and colleagues analyzed fecal samples from non-small cell lung cancer (NSCLC) patients and grouped them according to their treatment response based on the Response Evaluation Criteria in Solid Tumors (RECIST). The researchers compared the gut microbiome compositions of the different groups and found that the responders had a higher abundance of Bifidobacterium (64). The researchers then selected four Bifidobacterium strains to test in a tumor mouse model (MC38 cell line). Starting 14 days before tumor implantation, the mice were treated with Bifidobacterium daily. The results showed that all Bifidobacterium strains delayed tumor growth, but only two strains (B.bif_K57 and B.bif_K18) exhibited synergistic anti-tumor effects when combined with PD-1 inhibitors. By comparing the immune cell populations in the tumors and spleens of mice treated with the synergistic strain (B.bif_K57) and the non-synergistic strain (B.bif_B06), the researchers found that the synergistic group had a higher number of immune-active cells, such as CD4^+^ T cells, CD8^+^ T cells, and NK cells, while Treg cells were fewer. Further confirmation by qPCR and flow cytometry showed that combining B.bif_K57 with the PD-1 inhibitor significantly increased the expression of immune-active cytokines, such as IFN-γ and IL-2, in the tumor, while reducing the expression of immune-suppressive cytokines like TNF-α and IL-10. These findings suggest that Bifidobacterium can enhance tumor immune activation induced by PD-1 inhibitors.

## 7 Bifidobacterium metabolism and the tumor immune microenvironment

Bifidobacterium can regulate the tumor immune microenvironment (TIME) through various metabolic pathways, enhancing anti-tumor immune responses and improving the effectiveness of immunotherapy. Its metabolic products, such as short-chain fatty acids (SCFAs) and extracellular polysaccharides (EPS), promote the maturation of dendritic cells (DCs), enhancing their antigen-presenting capability, thereby boosting the activation and cytotoxicity of CD8+ T cells (65). Furthermore, Bifidobacterium can induce tumor-associated macrophages (TAMs) to transition from the immune-suppressive M2 phenotype to the pro-inflammatory M1 phenotype, further enhancing anti-tumor immunity (62).

Regarding the gut-tumor immune axis, Bifidobacterium improves gut microbiota homeostasis, strengthens gut barrier function, and activates innate immune signaling pathways through pattern recognition receptors (such as TLR2/4), promoting the infiltration and functional recovery of T cells in the distal tumor microenvironment (66). Additionally, Bifidobacterium can reduce the presence of immunosuppressive cells in the tumor microenvironment, including regulatory T cells (Tregs) and myeloid-derived suppressor cells (MDSCs), and decrease the secretion of inhibitory cytokines such as IL-10 and TGF-β, thereby alleviating the immune suppressive state and enhancing anti-tumor immune responses (67).

Recent studies have demonstrated that Bifidobacterium can significantly enhance the therapeutic effect of immune checkpoint inhibitors (ICIs). The mechanism likely involves boosting T cell activity, improving antigen presentation, and upregulating PD-L1 expression, making tumors more sensitive to PD-1/PD-L1 inhibitors (63). This finding provides a new strategy to overcome tumor resistance to immunotherapy. In conclusion, Bifidobacterium, by regulating immune cell function, improving the gut-tumor immune interaction, reducing immunosuppressive signals, and enhancing the response to immune checkpoint inhibition, offers important research directions and clinical potential for optimizing the tumor immune microenvironment, improving anti-tumor efficacy, and developing novel combination therapies.

## 8 Clinical applications of bifidobacterium

Recent evidence suggests that Bifidobacterium may enhance anti-tumor immune responses and improve the effectiveness of immunotherapy. It was previously hypothesized that the abundance of Bifidobacterium in colorectal cancer tissues might be correlated with tumor differentiation and the intensity of the immune response in colorectal cancer. In a subsequent study, Keisuke Kosumi and his team analyzed data from the molecular pathological epidemiology database of 1,313 cases of rectal and colon cancer. They measured the levels of Bifidobacterium DNA in cancer tissues using quantitative PCR and compared it with the presence of circular cells and other tumor characteristics. The relationship between Bifidobacterium levels, tumor differentiation, circular cell prevalence, and extracellular mucin was then assessed. The results indicated that the abundance of circular cells in colorectal cancer tissues was positively correlated with Bifidobacterium abundance, suggesting that Bifidobacterium may influence tumor characteristics or serve as an indicator of mucosal barrier dysfunction in colorectal cancer (68).

Additionally, Sumanta K. Pal’s research team was the first to demonstrate that probiotic oral medications can improve gut microbiome homeostasis in cancer patients and enhance their response to immunotherapy (69). The study revealed that in patients with metastatic renal cell carcinoma (mRCC), the addition of the probiotic drug CBM588 during treatment with PD-1 inhibitor nivolumab and CTLA-4 inhibitor ipilimumab significantly improved the median progression-free survival (PFS) and response rate. The study included 30 treatment-naive mRCC patients, who were randomly assigned in a 2:1 ratio to the CBM588 + O drug + Y drug group and the O drug + Y drug group. The median PFS in the O drug + Y drug group was 2.5 months, with a partial response rate of 20%. In contrast, the CBM588 + O drug + Y drug group had a median PFS of 12.7 months and a partial response rate of 58%. This indicates that the combination of the probiotic CBM588 oral medication significantly extended the median PFS in mRCC patients from 2.5 months to 12.7 months, a nearly 400% increase, and improved the partial response rate from 20% to 58%, a nearly threefold increase.

In another 2023 study, the authors investigated the association between postoperative liver function recovery (LFR) and the gut microbiome. They prospectively recruited 123 patients with hepatocellular carcinoma (HCC) and collected their stool samples before and after surgery for 16S rRNA amplicon sequencing. The results showed that Bifidobacterium longum (B. longum) was the most contributing genus (70). To further validate the beneficial effects of B. longum on recovery in humans, the authors conducted an open, randomized, placebo-controlled clinical trial. The intervention group (BL) received a probiotic mixture containing B. longum (at least 1.0×10^7^ CFUs) during the perioperative period, while the control group (CON) received no probiotic mixture or other probiotic supplements. A total of 169 patients completed the trial, and the results showed that the BL group had significantly fewer patients with delayed recovery postoperatively compared to the CON group. Additionally, the BL group had a significantly shorter postoperative hospital stay (average of 8.34 days) compared to the CON group (average of 9.67 days). Moreover, the CON group exhibited significantly shorter one-year survival, with similar trends observed for two-year survival. These findings confirm the promotive effect of B. longum on liver function recovery in HCC patients post-surgery.

## 9 Advantages of bifidobacterium in safety and targeting

Currently, an increasing number of microorganisms are used for immune activation. **Table 1** summarizes the differences between Bifidobacterium, Salmonella, and Listeria in terms of safety, tumor targeting, and immune activation effects.

**Table 1:** Comparison of bifidobacterium, salmonella, and listeria in cancer therapy.

Indicator	Bifidobacterium	Salmonella	Listeria
Tumor Targeting Ability	High, preferentially targets hypoxic tumor microenvironment	High, can actively invade tumors	High, can invade host cells
Safety	Very high, no significant toxicity	Low, requires detoxification modification	Low, potential zoonotic risks
Host Immune Activation	Moderate, can induce anti-tumor immunity	High, can trigger strong immune responses	High, can induce cellular immunity
Genetic Engineering Difficulty	Moderate, genetic manipulation is relatively mature	Low, can easily knock out toxic genes	High, manipulation is more complex
Research Application Status	More preclinical studies, some enter clinical trials	Some strains enter clinical trials	Mainly in experimental research phase

## 10 Challenges of bifidobacterium in clinical translation

As research progresses, particularly in the interactions between Bifidobacterium and the tumor microenvironment, along with advancements in genetic engineering that allow for the customization of bacterial strains, the potential applications of Bifidobacterium in cancer therapy could significantly expand. However, several challenges remain to be addressed, such as the stability of engineered bacteria (risk of plasmid loss), safety (immune activation and toxicity), and dose optimization. The risk of plasmid loss in engineered bacteria is primarily influenced by factors such as plasmid stability, selection pressure, the metabolic burden of the host bacterium, culture conditions, and host strain characteristics. For example, high-copy or large-size plasmids are more prone to loss due to increased metabolic burden, and in the absence of antibiotic or nutrient selection pressure, the host bacteria may gradually eliminate plasmid-carrying cells. Additionally, factors such as prolonged cultivation, high temperatures, and shear forces can accelerate plasmid loss (71).

Regarding bacterial immune activation and safety, as bacteria are complex and feasible therapeutic agents, some uncontrollable mutations during bacterial proliferation may introduce potential toxicity, and their inherent virulence can lead to complex infections in immunocompromised cancer patients (72). Furthermore, dose optimization for bacterial therapies involves multiple key factors, including bacterial type, growth characteristics, host immune response, target site, and administration route, to ensure therapeutic efficacy while minimizing side effects. Optimization methods mainly include pharmacokinetics/pharmacodynamics (PK/PD) modeling to predict bacterial diffusion and clearance in the body, dose escalation trials, and animal model validation to determine the minimum effective dose (MED) and maximum tolerated dose (MTD).

## 11 Conclusion

Bifidobacterium, as a biological targeting agent, introduces an innovative strategy for cancer therapy. Its selective targeting ability makes it a valuable tool for enhancing the effectiveness of traditional treatment methods such as chemotherapy, radiotherapy, and immunotherapy. By serving as a delivery vehicle for therapeutic agents, Bifidobacterium can improve the targeting and efficacy of treatments while reducing side effects associated with off-target toxicity. Additionally, its inherent biosafety, precise targeting ability, and adaptability make Bifidobacterium an exciting area of research in oncology. Its application may offer patients more personalized and targeted treatment options, potentially resulting in better therapeutic outcomes with fewer side effects. Future research is crucial for advancing our understanding of the clinical applications of Bifidobacterium.
